# Rupture of Abdominal Aortic Aneurysm After Bevacizumab Treatment for Colorectal Cancer

**DOI:** 10.7759/cureus.87776

**Published:** 2025-07-12

**Authors:** Naoki Uemura, Hirofumi Saitoh, Junji Shiotsuka, Shigehiko Uchino, Shinshu Katayama

**Affiliations:** 1 Department of Anesthesiology and Critical Care Medicine, Jichi Medical University Saitama Medical Center, Saitama, JPN

**Keywords:** abdominal aortic aneurysm repair, abdominal aortic rupture, bevacizumab toxicity, colorectal cancer, emergent surgery, gastrointestinal oncology

## Abstract

Bevacizumab, a humanized monoclonal antibody against vascular endothelial growth factor (VEGF), is used in combination with chemotherapy for various malignancies, including metastatic colorectal cancer. While effective, bevacizumab can inhibit normal blood vessel growth, leading to cardiovascular side effects not typically associated with conventional chemotherapy. We report a rare case, from an international perspective, of a 73-year-old man with a history of gastric cancer and newly diagnosed metastatic colorectal cancer complicated by a pre-existing abdominal aortic aneurysm (AAA) measuring 52 mm. The aneurysm was initially managed conservatively, as the multidisciplinary team (MDT) and the patient agreed to prioritize chemotherapy despite the known rupture risk, given his wish to avoid delaying treatment for his cancer. After the diagnosis of colorectal cancer during chemotherapy, bevacizumab was added to his regimen. He developed a rupture of the AAA two days after the fourth dose. Emergent open surgical repair was successfully performed without wound healing complications. This case highlights the potential risk of large-vessel complications associated with bevacizumab, especially in patients with known vascular anomalies. Careful imaging assessment and monitoring are imperative when considering bevacizumab for patients at risk of aortic rupture. In selected cases, prophylactic measures such as preemptive aneurysm repair should be contemplated to optimize safety.

## Introduction

Angiogenesis, the formation of new blood vessels, is essential for tumor growth and progression, and targeting this process has become an important strategy in cancer therapy. Vascular endothelial growth factor (VEGF), particularly its predominant isoform, vascular endothelial growth factor A (VEGF-A), is a key molecular driver of this process. As a result, VEGF-A has become a major therapeutic target in efforts to block tumor vascularization and thereby suppress tumor progression by limiting its blood supply. Several anti-angiogenic drugs have been approved for treating various cancers, including metastatic colorectal cancer, renal cell carcinoma, non-squamous non-small cell lung cancer, hepatocellular carcinoma, cervical cancer, and ovarian cancer [1].

Bevacizumab (Avastin®), the first anti-angiogenic drug, is a humanized monoclonal antibody against VEGF-A. It was initially approved for the treatment of previously untreated metastatic colorectal cancer in combination with chemotherapy. Bevacizumab selectively binds to VEGF-A, inhibiting its interaction with receptors on endothelial cells, thereby disrupting blood vessel recruitment and growth [2]. According to the National Comprehensive Cancer Network (NCCN) Clinical Practice Guidelines in Oncology (Colon Cancer, Version 1.2024), bevacizumab remains an important treatment option in combination with chemotherapy for patients with metastatic colorectal cancer [3].

As VEGF also plays an important role in angiogenesis of normal tissues, its blockade can inhibit the proliferation of normal blood vessels [4]. Consequently, anti-VEGF drugs, including bevacizumab, can cause adverse cardiovascular side effects not typically associated with conventional cytotoxic chemotherapeutic agents, including arterial thromboembolic events, coronary artery disease, and hypertension [5]. 

Moreover, previous research has suggested that VEGF pathway inhibition may impair vascular integrity through mechanisms such as endothelial dysfunction, smooth muscle cell loss, and extracellular matrix degradation. These changes may potentially weaken the aortic wall, raising concern for complications such as aneurysm formation or rupture in susceptible individuals [6]. However, to our knowledge, only one case report in English exists on the use of anti-VEGF agents in patients at risk of aortic rupture, as discussed later.

In this report, we present a rare case of a patient with metastatic colorectal cancer and a pre-existing abdominal aortic aneurysm (AAA), who developed a fatal rupture of the AAA following bevacizumab administration. This case underscores the importance of careful assessment and monitoring when considering bevacizumab for patients with known vascular anomalies.

## Case presentation

A 73-year-old man presented with anorexia and weight loss. His medical history was significant for hypertension, peripheral artery disease, and diabetes mellitus. Initial evaluations included upper gastrointestinal endoscopy, which revealed a type 4 gastric tumor; biopsy confirmed poorly differentiated adenocarcinoma. Computed tomography (CT) scans showed gastric wall thickening and metastases to the liver, peritoneum, and lymph nodes, leading to a diagnosis of advanced type 4 gastric cancer, with suspected peritoneal dissemination. A multidisciplinary team (MDT) decided to initiate chemotherapy with mFOLFOX6 (oxaliplatin, levoleucovorin, and fluorouracil) for gastric cancer.

**Figure 1:**
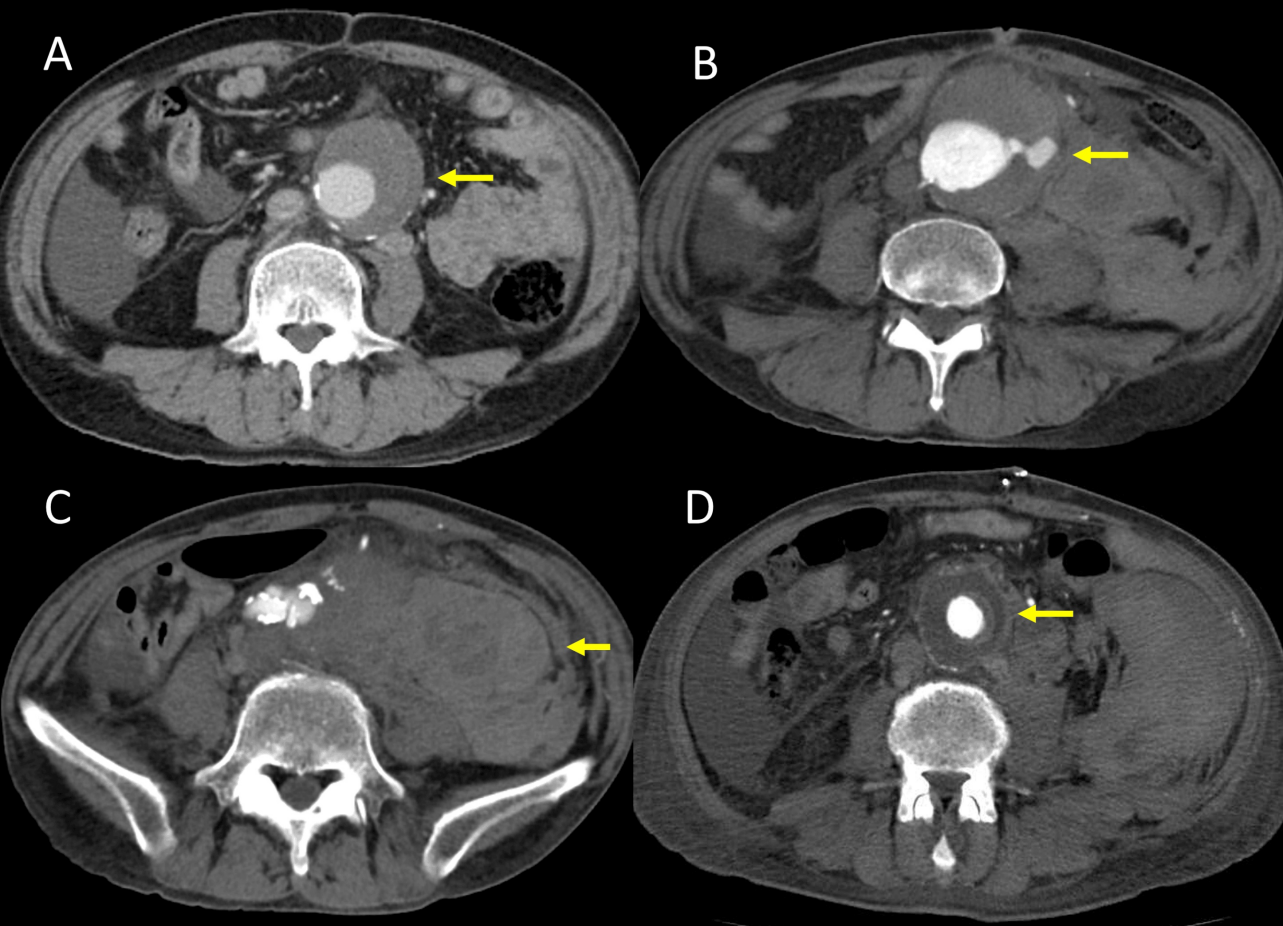
Comparison of pre-treatment and post-rupture axial CT findings. (A) Pre-treatment axial CT image showing an infrarenal abdominal aortic aneurysm measuring 52 mm. (B) Axial CT image after rupture demonstrating an enlarged aneurysm measuring 70 mm. (C) Axial CT image after rupture revealing a large retroperitoneal hematoma. (D) Postoperative axial CT image showing a 22×11 mm aorto-uni-iliac Dacron graft. Arrows in each panel indicate the site of aortic aneurysm (A, B), retroperitoneal hematoma (C), and graft placement (D).

**Figure 2:**
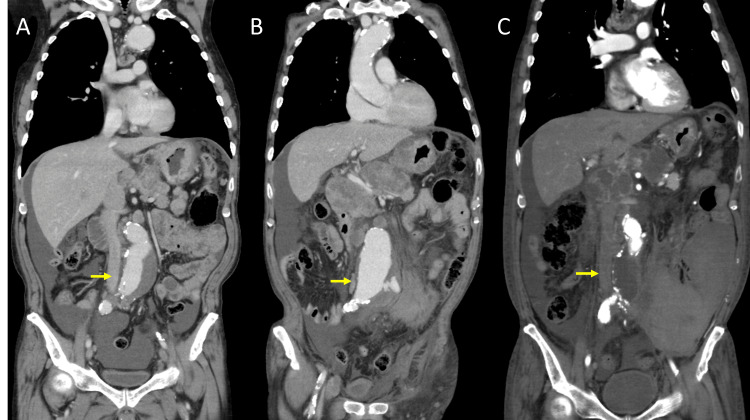
Comparison of pre-treatment and post-rupture CT findings. (A) Pre-treatment coronal CT image showing abdominal aortic aneurysm. (B) Coronal CT image after rupture demonstrating an enlarged aneurysm measuring 70 mm. (C) Postoperative coronal CT image showing a 22×11 mm aorto-uni-iliac Dacron graft, consistent with that shown in Figure 1D. Arrows in each panel indicate the location of the aortic aneurysm or the aortic graft.

Simultaneously, CT angiography revealed thoracic and abdominal aortic aneurysms, with the thoracic aneurysm measuring 48 mm and the AAA measuring 52 mm in diameter (Figures 1A, 2A). Generally, surgical repair is recommended for abdominal aortic aneurysms (AAAs) larger than 50 mm in men due to the higher risk of rupture [7]. However, the multidisciplinary team (MDT) and the patient agreed to prioritize the initiation of chemotherapy. The patient understood the potential risk of rupture but wished to avoid delaying treatment for his advanced gastric cancer. Consequently, conservative management was chosen, and we increased the patient’s amlodipine dose from 5 mg to 10 mg daily, which he had been taking prior to treatment. No specific advance directives, such as Do-Not-Attempt-Resuscitation (DNAR), were established regarding rupture-related events at that time. 

In addition, CT imaging also indicated that the left common iliac artery was chronically occluded. The ankle-brachial index (ABI) measurements were 1.04 on the right and 0.58 on the left. Despite the reduced ABI, the patient did not report claudication or other symptoms of limb ischemia. Because the occlusion was asymptomatic and distal perfusion was considered adequate, no specific intervention was performed for the left common iliac artery. 

**Figure 3:**
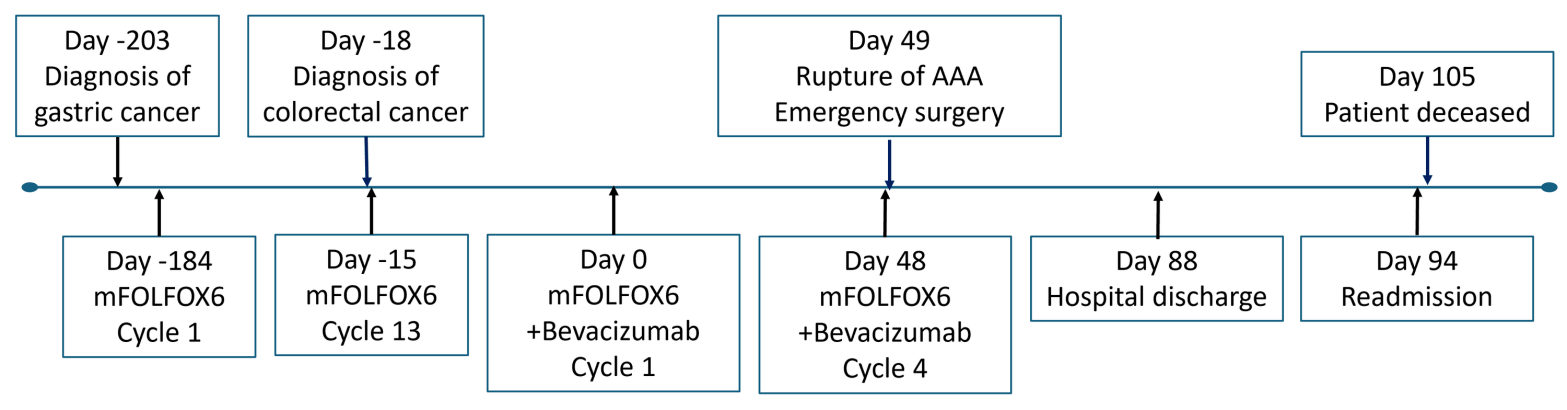
Timeline depicting the major events during the patient’s disease course as described in the text. mFOLFOX6: oxaliplatin, levoleucovorin, fluorouracil); AAA: abdominal aortic aneurysms

The patient underwent 13 cycles of mFOLFOX6 therapy for his gastric cancer (the chronological sequence of treatment and associated events is summarized in Figure 3 to provide a visual overview of the clinical course). After four months of treatment, repeat enhanced CT scans revealed a newly developed rectal lesion. Colonoscopy with biopsy confirmed rectal adenocarcinoma. Histopathology showed the gastric tumor was poorly differentiated (por > tub2), while the rectal tumor was well to moderately differentiated (tub1-2), supporting that this was a second primary malignancy rather than metastasis. Given the short interval, this was classified as synchronous cancer [8].

At that time, previously noted metastatic lesions, including hepatic, peritoneal, and pelvic lymph node involvement, were considered to be at least partly attributable to rectal cancer. CT findings demonstrated nodules in the rectovesical pouch and enlarged lymph nodes in the para-aortic, pelvic, and perirectal regions. Although formal clinical staging was not explicitly recorded, the rectal cancer was considered clinically consistent with stage IV disease (likely cT3-4N1-2M1c). Based on this clinical assessment, bevacizumab was added to the chemotherapy regimen.

Two days after receiving the fourth dose of bevacizumab (39 days after the first administration), the patient experienced a sudden onset of left lower abdominal pain and was brought to our hospital. On arrival, he was hypotensive and tachycardic, indicating hemodynamic instability. Urgent CT angiography demonstrated rupture of the AAA with retroperitoneal hemorrhage (Figures 1B, 1C, 2C). Given the life-threatening nature of the condition, surgical intervention was deemed necessary despite the associated risks, and emergent open surgical repair was selected over endovascular aneurysm repair (EVAR), primarily due to anatomical considerations.

Under general anesthesia, a full midline abdominal incision extending from the upper to the lower abdomen was made, and the peritoneal cavity was entered. A large amount of ascites, likely related to peritoneal carcinomatosis, was observed, and a significant retroperitoneal hematoma was identified. After gaining proximal and distal control of the aorta, the aneurysmal segment was resected. Reconstruction was performed using a bifurcated J-graft (22×11 mm) made of Dacron. As previously mentioned, the left common iliac artery was occluded due to atherosclerotic obstruction, and the patient was asymptomatic. Given that this was an emergency operation for a ruptured AAA and that the primary goal was life-saving intervention, revascularization of the left side was not performed. The left limb of the bifurcated graft was sutured closed, ligated, and resected. Distal anastomosis was completed to the right common iliac artery (Figures 1D, 2C). After completion of the anastomosis, Doppler ultrasound confirmed pulsatile flow in the left dorsalis pedis artery, suggesting sufficient collateral perfusion. The procedure was completed successfully without intraoperative complications.

Postoperatively, the patient was transferred to the intensive care unit (ICU). He was extubated on postoperative day one and remained in the ICU for three days. A postoperative CT angiogram performed on day 10 confirmed successful vascular repair with no evidence of graft-related complications. After transfer to the general ward, he developed a high fever; however, there were no signs of surgical site infection or delayed wound healing. Blood cultures were negative, and imaging studies did not reveal a specific source of infection. Empiric antibiotic therapy with meropenem was initiated, leading to the resolution of the fever within a day. Given its late onset and quick resolution, the fever was unlikely to be surgical or neoplastic in origin, and its cause remained unclear. Throughout his hospitalization, careful monitoring for wound healing complications was conducted due to the recent bevacizumab therapy, but no such complications were observed. The patient was discharged home 38 days after surgery in stable condition.

However, six days after discharge, he was readmitted with general weakness and clinical deterioration related to the progression of his gastric cancer. Despite supportive care, his condition worsened, and he passed away shortly thereafter due to advanced malignancy.

## Discussion

Colorectal cancer is the third most common cancer and the second leading cause of cancer-related deaths worldwide [9]. In patients with early-stage colorectal cancer, surgical resection followed by adjuvant chemotherapy is the standard treatment. However, because colorectal cancer often presents symptoms only after it has advanced, a significant proportion of patients are diagnosed at an advanced stage, making radical surgical treatment unfeasible. Consequently, the development of innovative and effective therapies is essential, with tumor angiogenesis emerging as a critical target in cancer treatment. 

Bevacizumab (Avastin®) was initially approved for the treatment of previously untreated metastatic colorectal cancer and later received expanded indications. Ferrara et al. introduced bevacizumab, the first anti-angiogenic drug, to address this target [10].

While these medications have demonstrated efficacy in late-stage and metastatic cancers [11,12], they are associated with side effects such as bleeding, arterial thromboembolic events, coronary artery disease, hypertension, wound healing complications, and, more rarely, gastrointestinal perforation and fistulas [13,14]. These adverse effects are thought to result from the inhibition of angiogenesis in normal tissues.

An association between bevacizumab and AAA rupture has not been clearly established, and only one other case report in English exists of aortic rupture occurring during anti-VEGF therapy [15]. A patient who presented with a bevacizumab-induced abdominal aortic rupture and the formation of an aorto-caval fistula was described in the report. While causality is not confirmed, our case is hypothesis-generating and suggests a possible association between bevacizumab and AAA rupture, calling for further research on this issue. VEGF plays a crucial role in normal tissue angiogenesis, including vascular maintenance and repair of vascular injury. Inhibiting VEGF can impair revascularization and normalization of immature or abnormal blood vessels, potentially leading to the deterioration of arterial aneurysms [4]. Mechanistically, VEGF inhibitors, including bevacizumab, have been associated with major vascular complications such as aortic dissection, and it is possible that inhibition of the VEGF pathway may compromise aortic wall integrity [16]. VEGF signaling is essential for endothelial function and smooth muscle cell proliferation, both of which are important for maintaining the extracellular matrix. Inhibition of this pathway may lead to weakening of the aortic wall. Experimental models have shown that VEGF inhibition can alter aortic wall remodeling and potentially contribute to the development of aortic dissection [17]. It is plausible that bevacizumab administration could contribute to aortic aneurysm rupture through similar mechanisms. Hypertension is a well-known side effect of bevacizumab and is also a significant risk factor for AAA rupture [18]. Bevacizumab may also play a role in the rupture of an AAA in this regard. In our case, the rupture occurred two days after the fourth dose of bevacizumab, 39 days after the initial administration. Considering bevacizumab's half-life of approximately 20 days [19], cumulative dosing could have led to increased serum concentrations, enhancing its side effects and contributing to the rupture.

These factors collectively suggest that bevacizumab carries potential risks for large-vessel complications and should be used with great caution in patients with AAAs. A thorough vascular assessment, including imaging studies like CT angiography, is essential before initiating bevacizumab therapy in such patients. According to clinical guidelines, abdominal aortic aneurysms measuring ≥50 mm warrant imaging surveillance every 3-6 months, and prophylactic intervention such as EVAR may be considered based on individual risk assessment [7].

Wound healing complications are another side effect of VEGF inhibitors, posing significant challenges during surgical treatments. Particularly, patients with colorectal cancer are reported to be more susceptible to wound healing complications caused by bevacizumab [20]. When planning elective surgery, it is recommended to wait approximately 40 days after the final administration of bevacizumab before proceeding [21]. In our case, emergency surgery was necessary, but fortunately, we were able to complete the procedure without any wound healing complications. Careful monitoring of the surgical wound is especially important in postoperative management during bevacizumab use.

Bevacizumab has been shown to improve progression-free survival but does not significantly enhance overall survival in metastatic colorectal cancer [12]. In this case, considering its cardiovascular toxicity and the side effects of delayed wound healing, the risks of bevacizumab may have outweighed its potential benefits, especially given the patient's pre-existing large AAA. A careful individualized risk-benefit assessment is paramount in such complex cases.

The current case report has limitations. As a single case, it cannot establish causality between bevacizumab and AAA rupture. However, the temporal relationship and known mechanisms of VEGF inhibition warrant strong consideration of this association. Further studies, potentially through registries or larger observational cohorts, are needed to better quantify the risk of AAA rupture in patients receiving bevacizumab, particularly those with pre-existing aneurysms. Establishing such registries or incorporating real-world data collection could help identify at-risk populations and guide safer treatment strategies.

In conclusion, when a patient has a vascular complication, a systemic exploration of vascular complications is imperative before the administration of bevacizumab. If bevacizumab is to be administered, utmost caution and regular close follow-up are required. Additionally, if there is evidence of disease progression, like the expansion of an AAA, not only intensification of antihypertensive therapy but also preventive surgery, such as endovascular repair, might be considered.

## Conclusions

Bevacizumab is an effective treatment for metastatic colorectal cancer; however, in patients with pre-existing vascular conditions such as AAAs, therapeutic decisions must carefully weigh the oncologic benefits against the potential risk of severe vascular complications. Individualized risk assessment is essential to guide safe treatment planning in such high-risk cases. This case suggests a possible association between bevacizumab therapy and AAA rupture, highlighting the importance of thorough vascular assessment before initiating treatment. For patients with large AAAs, prophylactic aneurysm repair should be strongly considered before commencing bevacizumab, or alternative anticancer therapies with a more favorable vascular safety profile should be explored. If bevacizumab is deemed necessary in high-risk patients, intensive blood pressure management and regular aortic surveillance are crucial.
